# Needs Assessment and Tailored Training Pilot for Emergency Care Clinicians in the Prehospital Setting in Rwanda

**DOI:** 10.5811/westjem.18698

**Published:** 2024-11-20

**Authors:** Naz Karim, Jeanne D’Arc Nyinawankusi, Mikaela S. Belsky, Pascal Mugemangango, Zeta Mutabazi, Catalina Gonzalez Marques, Angela Y. Zhang, Janette Baird, Jean Marie Uwitonze, Adam C. Levine

**Affiliations:** * Brown University Alpert Medical School, Department of Emergency Medicine, Providence, Rhode Island; † Service d’Aide Médicale Urgente, Kigali, Rwanda; ‡ NYU Grossman School of Medicine, New York, New York; § University Teaching Hospital, Kigali, Rwanda; ∥ University of Rwanda, Department of Anaesthesia, Emergency Medicine and Critical Care, Kigali, Rwanda; ¶ Brigham and Women’s Hospital, Department of Global Emergency Care and Humanitarian Studies, Boston, Massachusetts; # University of Washington, Department of Pediatrics, Seattle, Washington

**Keywords:** trauma, training, emergency, prehospital, education

## Abstract

**Background:**

In low- and middle-income countries (LMIC), 45% of deaths could be addressed by implementation of an emergency medical services (EMS) system. Prehospital care is a critical component of EMS worldwide, and basic, affordable training has been shown to improve EMS systems. However, patient outcome impact is unclear. In this study we aimed to assess the current state of prehospital care in Kigali, Rwanda, through a needs assessment, focused training intervention, and analysis of current practices and patient outcomes.

**Methods:**

We identified 30 clinicians through the prehospital medical command office and included them in the study. A prospective, nonrandomized, interrupted time-series approach was used. Data collected through closed- and open-ended questionnaires included age, sex, training, and knowledge assessment. We used the data to create a tailored, 18-hour training after which immediate and 11-month post-tests were administered. Linked prehospital and hospital care datasets allowed for evaluation of patient outcomes and prehospital process indicators that included training skill application, airway intervention, intravenous fluid administration, and glucose administration.

**Results:**

Of 30 clinicians, 18 (60%) were female, 19 were nurses, and 11 were nurse anaesthetists. Median age was 36, and median years providing care was 10 (IQR 7–11). Twenty-four (80%) participants completed immediate and post-test assessments. Mean knowledge across 12 core skills significantly improved from a pre-test mean of 59.7% (95% confidence interval [CI] 42.2–77.20) to a post-test mean of 87.8% (95% CI 74.7–100). At 11 months post-training, the score improvement maintained, with a mean score of 77.6% (95% CI 59.2–96.8). For patient outcomes, the total sample size was 572 patients; 324 of these patients were transported to the ED during the pre-training period (56.4%), while 248 were transported post-training. Prehospital oxygen administration for patients with a saturation level of <95% significantly increased pre- to post-training (66.7% to 71.7%; Δ = 5.0%; Δ95% CI 1.9,−8.1%). No significant changes were noted in patient treatment outcomes or other process indicators due to small sample sizes.

**Conclusion:**

This study provides insights on Rwandan EMS and demonstrates that a tailored intervention targeting education on prehospital process indicators has positive impacts on clinician knowledge and practice.

Population Health Research Capsule What do we already know about this issue?

*In low- and middle-income countries, 45% of deaths could be addressed by emergency medical service (EMS) system implementation*.

What was the research question?

*How does am EMS training intervention impact current practices and patient outcomes?*

What was the major finding of the study?

*The use of oxygen for patients showed a significant increase post-training intervention (pre 66.7%, post 71.7%; Δ = 5.0%; Δ 95% CI 1.9–8.1%).*

How does this improve population health?

*Basic and affordable EMS training interventions can improve clinical medical knowledge and clinical processes.*

## INTRODUCTION

### Background

Emergency medical systems (EMS) provide populations with acute care for a diverse set of diseases spanning the spectrum of communicable infections, non-communicable diseases, obstetric and paediatric emergencies, as well as traumatic injuries.^1^ The 2003 World Health Report drew attention to the increasing burden of non-communicable disease, chronic diseases, and trauma in low- and middle-income countries (LMIC)^2^ and has called for “rapid and sustainable expansion of emergency treatments” globally.^3^ Several studies, supplemented by reports from the World Health Organization (WHO), emphasize the importance of improving access to emergency health services to reduce morbidity and mortality specifically in LMICs.^1^ ^–^ ^11^ An estimated 45% of deaths and 36% of disability-adjusted life years (DALY) in LMICs could be addressed by the implementation of emergency care systems.^4^ ^,^ ^5^ ^,^ ^6^

Prehospital care is a critical component of emergency care worldwide^1^. A systematic review of trauma systems by Mann et al showed consistent survival benefits following inexpensive interventions, such as organization and trauma system planning.^7^ Survival benefits from addition of basic and affordable training to EMS have also been noted by health experts.^12^ ^,^ ^13^ In resource-limited settings, prehospital training has shown success in improving clinician knowledge, although impact on clinician practice and patient outcomes is unclear.^14^ ^,^ ^15^ Research on in-hospital emergency medicine training in LMICs is more robust, showing feasibility for such programs to improve patient and clinical outcomes as well as clinician knowledge.^16^ ^,^ ^17^ Still, even for in-hospital programs, there remains a need for further research regarding their impact.^16^ Further, existing literature on prehospital training in LMICs most often focuses on first responders who may not have formal trauma and emergent care training or who do not solely work in the prehospital setting.^18^ ^–^ ^21^

In Rwanda, the Ministry of Health (MOH) has made great strides in providing access to healthcare by rebuilding its health infrastructure in the aftermath of the 1994 genocide. To address prehospital care access, the MOH implemented an emergency medical ambulance system in 2007, named Service d’Aide Medicale Urgente (SAMU).^22^ The SAMU prehospital professionals at the Centre Hospitalier Universitaire de Kigali (CHUK) are the sole providers of prehospital emergency care in Rwanda. Ambulances are equipped with oxygen tanks, intravenous fluids, supplies for peripheral line placement, medications, and defibrillators, and are staffed by nurses and nurse anaesthetists. A1 registered nurses with an advanced diploma have completed three years of post-secondary education while A0 nurses have a bachelor’s degree with four years of post-secondary education and are capable of intubating patients.

Along with inexpensive training and trauma planning interventions, survival benefits associated with EMS are based largely on the identification of key performance indicators to improve the quality of patient care.^1^ ^,^ ^13^ ^,^ ^23^ Thus, the Rwandan Human Resources for Health Strategic Plan recognized the need for nurse training programs that develop prehospital skills, while also strengthening ongoing mentoring and monitoring, especially as the standard nursing curriculum only offers in-hospital care training.^22^ Despite the increased capacity of the Rwandan prehospital system, there is a paucity of information about the needs of prehospital professional training as well as limited information about current practices of prehospital management and its relationship to patient outcomes. To our knowledge this is the first study that aims to understand the needs of prehospital care professionals, and the impact of a novel prehospital professional training on clinical responsiveness and patient outcomes.

## METHODS

### Study Design

The study was conducted in multiple phases. In the first phase we analysed prehospital care needs and emergency care knowledge. The second phase was a training intervention designed from the results of phase 1. In the third phase we assessed data on prehospital process indicators and patient outcomes before and after the training intervention, as well as prehospital professionals’ pre- and post-test scores, to evaluate the impact of the training, using a prospective, quasi-experimental, non-randomized, interrupted time series design.

### Study Setting

The training intervention was held at the Kigali Institute of Science and Technology (KIST) in May 2017. and we used data from the prehospital database at the University Teaching Hospital in Kigali (UTH-K) to analyse prehospital process indicators and patient outcomes. The study was approved by the Lifespan Institutional Review Board and the Institutional Review Board and Ethics Committee of UTH-K.

### Participants

All 30 prehospital care professionals at the UTH-K were included in the needs assessment and training intervention. We identified the study population by a list of prehospital professionals as reported by the human resources department and confirmed with the director of the ambulance services. A non-randomized approach was taken to minimize interruption in participant workflow. We excluded ambulance drivers as well as EMS administrative staff at the call centres not directly involved in patient care.

We identified the patient study population sample through paper, prehospital ambulance records. A trained research assistant linked prehospital paper run sheets to emergency department (ED) health records. The linkage was performed through querying OpenClinic, an open-source, integrated hospital information management system (OpenClinic GA, San Diego, CA). Patient names, date of birth, and addresses found in OpenClinic were matched to the SAMU database to confirm identity. The eligible analytical sample included all patients transported to the UTH-K ED by SAMU between December 1, 2017–June 30, 2018. These dates are inclusive of four months of pre-training and three months post-training. We excluded paediatric patients with medical chief complaints and obstetric patients as they are transferred to their respective emergency centres.

### Data Measurement

The needs assessment and pre-test data were collected using a closed- and open-ended questionnaire. The survey consisted of three parts: 1) demographic data, including age, sex, preferred language, and information about prehospital professional background; 2) needs assessment on prior 12 months training received on 12 core clinical skills; and 3) pre-test questions assessing clinical knowledge about and confidence in performing these 12 skills. We used the information collected from this instrument for the creation of an educational intervention deemed to be locally appropriate and based on the region’s prehospital needs.

The EMS professionals attended a 17-hour educational intervention administered over two days. A two-day course was preferred by the local team to limit interruption of care to patients. The course structure included didactic lectures, team discussions, simulation exercises, and pre- and post-tests at the KIST simulation centre. The training intervention focused on pre-identified skills for improvement, based on the needs assessment. These skills are, therefore, inclusive of patient presentations that most often go unrecognized or untreated in a prehospital setting, as well as clinical interventions that are associated with decreased prehospital professional confidence. The efficacy of the training intervention was evaluated by an immediate post-test and 11-month post-test determining the knowledge gain and change in report of confidence in performing 12 core clinical skills (Table 1).

**Table 1.** Prior 12-month core skill training experience of prehospital professionals in Rwanda.

Core skill	I do not know what this means n (%)	No training n (%)	1–5 hours n (%)	6–10 hours n (%)	More than 11 hours n (%)
a. Taking vital signs	0	19 (65.5)	3 (10.4)	1 (3.4)	6 (20.7)
b. Placing an Intravenous line	0	19 (65.5)	2 (6.9)	1 (3.4)	7 (24.1)
c. Placing an Intraosseous access	0	27 (93.1)	0	1 (3.4)	1 (3.4)
d. Checking glucose	0	15 (57.7)	3 (11.5)	1 (3.9)	7 (26.9)
e. Performing the trauma ABC evaluation	1 (3.5)	10 (34.5)	3 (10.3)	6 (20.7)	9 (31.0)
f. Intubation	0	11 (64.7)	1 (5.9)	1 (5.8)	4 (23.5)
g. Immobilizing a trauma patient	0	12 (44.4)	2 (7.4)	5 (18.5)	8 (29.6)
h. Performing needle decompression	0	18 (64.3)	4 (14.3)	4 (14.3)	2 (7.2)
i. Performing CPR	0	3 (15.8)	0	6 (31.6)	10 (52.6)
j. Performing defibrillation	0	18 (64.3)	2 (7.1)	3 (10.7)	5 (17.9)
k. Splinting a fracture	0	16 (59.3)	3 (11.1)	0	8 (29.6)
l. Injecting aubcutaneous epinephrine	0	21 (72.4)	4 (13.8)	1 (3.5)	3 (10.3)

*CPR*, cardiopulmonary resuscitation.

To collect data regarding implementation of these learned skills, we used a restrictive sampling frame. Key process indicators were selected based on elements of EMS care that are both responsive to system improvements and achievable in low-resource settings. An emphasis was placed on interventions with a high probability of decreasing medically preventable death or disability due to trauma. Outcomes for analysis thus included the following: use of IV fluid resuscitation for hypotensive patients (blood pressure less than 90/60 millimeters of mercury) or patients with burn injury; administration of oxygen to hypoxic patients (oxygen less than 95%); and administration of glucose to hypoglycaemic patients (glucose less than 60 milligrams per decilitermg/dl).

Patient outcome data extracted from hospital records included initial vital signs, diagnosis, hospital length of stay, and condition upon discharge. The extracted data was de-identified and entered into a secured RedCap database (Research Electronic Data Capture, Vanderbilt University, Nashville, TN) hosted at Lifespan in Providence, Rhode Island.

### Statistical Methods

We analyzed pre- and post-tests for change in knowledge. Descriptive and inferential statistics were conducted, with median values and interquartile range (IQR) reported. Conducted statistical tests included the Fisher exact test for differences in proportions, the Wilcoxon rank sum test for tests of medians, and paired *t*-tests for differences in knowledge and confidence. Yes and no binary variables for prehospital clinical process indicators were coded as 1 or 0, respectively. The outcomes for fluid administration for patients with burns and those with hypotension were combined, due to the small numbers of patients (seven) with burns. Additionally, ED patient care outcomes pre- and post-training intervention were analysed. We conducted statistical analysis was conducted using SAS statistical software v 9.4 (SAS Institute Inc, Cary, NC).

## RESULTS

### Participants and Descriptive Data

Of the 30 prehospital clinicians, 18 (60%) were female, 19 were nurses, and 11 were nurse anaesthetists. The median age was 36 years and median time providing care was 10 years (interquartile ratio 7–11). Twenty-four participants (80%) completed the training intervention and an immediate post-test. Sixteen participants (53.3%) completed both the immediate and 11-month post-tests.

### Needs-Assessment Results

A needs assessment was conducted prior to training. Table 1 shows the frequency of prior past-year training reported by participants. Across the 12 core skills, ‘no training’ was most frequently reported for placing an intraosseous access (IO) (93.1%), injecting subcutaneous epinephrine (72.4%), taking vital signs (65.5%), and placing an IV line (65.5%). More than 11 hours of training was most often reported for performing cardiopulmonary resuscitation (52.6%), performing trauma ABC evaluation (31%), immobilizing a trauma patient (29.6%), and splinting a fracture (29.6%). Pre-intervention, 15 participants (50%) reported wanting more training on trauma skills (bleeding control, IV and IO line placement, use of oxygen masks, understanding vital signs).

Correspondingly, median confidence in performing these 12 core skills varied at the pre-training needs assessment (Table 2). Using the Wilcoxon rank-sum test with paired comparisons between the three assessments of confidence, we found there were significant increases in median confidence in placing an IO (pre to immediate post *P* = .02; pre to 3-month post *P* < .01); and performing needle decompression (pre to immediate post *P* = 0.0002, pre to 11-month post *P* = 0.003).

**Table 2.** Prehospital professionals’ confidence on Likert scale of 1–5 performing core skills.

Confidence in performing core skill	Median (IQR) pre training (1)	Median (IQR) post training immediate (2)	Median (IQR) post training 11 months (3)	Statistical comparison
a. Taking vital signs	5 (5,5)	5 (5,5)	5 (5,5)	1 vs. 2 = ns 1 vs. 3 = ns 2 vs. 3 = ns
b. Placing an intravenous line	5 (5,5)	5 (5,5)	5 (5,5)	1 vs. 2 = ns 1 vs. 3 = ns 2 vs. 3 = ns
c. Placing an intraosseous access	2 (2,3)	3 (2,4)	4 (3,4)	1 vs. 2 = 0.02 1 vs. 3 = 0.002 2 vs. 3 = ns
d. Checking glucose	5 (5,5)	5 (5,5)	5 (5,5)	1 vs. 2 = ns 1 vs. 3 = ns 2 vs. 3 = ns
e. Performing the trauma ABC evaluation	5 (4,5)	5 (5,5)	5 (5,5)	1 vs. 2 = ns 1 vs. 3 = 0.05 2 vs. 3 = ns
f. Intubation	3 (3,5)	4 (3,5)	5(4,5)	1 vs. 2 = ns 1 vs. 3 = ns 2 vs. 3 = ns
g. Immobilizing a trauma patient	5 (5,5)	5 (5,5)	5 (5,5)	1 vs. 2 = ns 1 vs. 3 = ns 2 vs. 3 = ns
h. Performing needle decompression	3 (3,4)	4 (4,5)	4 (4,5)	1 vs. 2 = 0.0002 1 vs. 3 = 0.003 2 vs. 3 = ns
i. Performing CPR	5 (4,5)	5 (4,5)	5 (5,5)	1 vs. 2 = ns 1 vs. 3= 0.04 2 vs. 3 = ns
j. Performing defibrillation	4 (3,4)	4 (3,4)	5 (4,5)	1 vs. 2 = ns 1 vs. 3 = 0.03 2 vs. 3 = 0.05
k. Splinting a fracture	5 (5,5)	5 (5,5)	5 (5,5)	1 vs. 2 = ns 1 vs. 3 = ns 2 vs. 3 = ns
l. Injecting subcutaneous epinephrine	4 (4,5)	5 (3,5)	5 (4,5)	1 vs. 2 = ns 1 vs. 3 = ns 2 vs. 3 = ns

ge; 1 = pre training, 2 = immediate post training, 3 = 11 months after training.

*CPR*, cardiopulmonary resuscitation; *IQR*, interquartile range.

### Knowledge Results

The mean pre-test correct knowledge score was 59.7% (95% confidence interval [CI] 42.2–77.20). The immediate post-test mean score was 87.8% (95% CI 74.7–100), and 11 months after training the mean test score was 77.6% (95% CI 59.2–96.8). There was a 56% (95% CI 36.2–75.8) relative increase in mean knowledge score that was maintained between the immediate and 11-month post-test. There was a significant gain in knowledge between pre-intervention and immediate post-test (*P* < .001), and this difference was significant between pre and 11-month post-test (*P* < 0.001) using paired t-tests comparing the survey administrations. The gain was significantly higher in the immediate post-test compared to the 11-month post-test (*P* = .02). Scores in the categories of patient assessment, respiratory intervention, and fluid therapy improved by 8%, 21% and 8%, respectively. All respondents reported that the training had both increased their medical knowledge and improved patient care, and all respondents requested further training.

### Patient Care Results

In total, 572 patients were transported to the ED for care during the study; 324 (56.4%) were transported during the pre-training period. The average patient age was 37 years (SD 15.8, range 15–96), and 148 (25.7%) were female, with no significant difference in average age (*P* = .08) or sex (*P* = .14) proportion pre- and post-training. Table 3 shows the frequency of clinical conditions of interest pre- and post-training and clinical outcomes. Using chi-square tests, we found no significant differences pre- to post-training across the three types of clinical presentation of interest (*P* = .68), ED disposition (*P* = .24), median hours spent in the ED (*P* = .64), or median length-of-hospital stay for those admitted (*P* = .85).

**Table 3.** Emergency department patient putcomes.

Clinical presentation n (%)	Pre training n = 324	Post training n = 248
Burn	5 (1.5)	2 (0.80)
Hypotension	15 (4.7)	13 (5.4)
Hypoxia	75 (23.2)	52 (20.7)
Hypoglycaemia	1 (0.30)	0
Clinical outcomes		
ED disposition	Admit = 154 (73.3) Transfer = 9 (4.3) Discharged = 39 (18.6) Died in ED = 8 (3.8) Died after admission = 18 (12.1)	Admit =142 (79.3) Transfer = 2 (1.1) Discharged = 33 (16.7) Died in ED = 5 (2.8) Died after admission = 15 (10.6)
Length of stay in ED (hours) Median (IQR)	24 (24,48)	24 (24,48)
Length of stay if admitted (days) Median (IQR)	3 (1,10)	3 (2,10)

*ED*, emergency department; *IQR*, interquartile ratio.

### Process Indicator Results

Table 4 details the frequency of prehospital clinical interventions before and after the training intervention. A review of data showed that IV fluid resuscitation was given to all indicated patients pre- and post-intervention, and that the use of oxygen for patients with a saturation level of < 95% showed a significant increase in proportion pre- to post-intervention (pre = 66.7%, post = 71.7%; Δ = 5.0%; Δ 95% CI 1.9–8.1%), as the 95% CI around the Δ does not include 0. Table 4 also shows this significant change in administration of oxygen for hypoxic patients over the pre- and post-intervention period.

**Table 4.** Prehospital clinical process indicators (prehospital professionals’ responsiveness).

Clinical presentation	Expected clinical intervention	Frequency administered pre training (%)	Frequency administered post training (%)
Burn and/or hypotension	Use of intravenous fluid resuscitation	20 (100)	15 (100)
Hypoxia (SpO_2_ < 95%)	Oxygen given	50 (66.7)	38 (71.7)
Hypoglycaemia	Glucose administered	0	0

## DISCUSSION

This study not only demonstrates statistically significant test improvements for a needs assessment-based, context-appropriate education intervention, but also explores prehospital process indicators and patient outcomes. The pre- to post-test improvements, especially regarding patient assessment, respiratory intervention, and fluid therapy, remained significant at the 11-month post-test, demonstrating long-term knowledge retention. Importantly, significant pre- to post-test increase in hypoxia responsiveness demonstrates actual implementation of learned skills. These concrete positive results are bolstered by statistically significant increases in prehospital clinical confidence, which occurred across multiple skills. These improvements are important, as self-confidence has been shown to enhance clinical competency and effective clinical decision-making.^24^ Additionally, participants noted positive responses to the training modules and even requested additional training, speaking to desirability and feasibility of such prehospital training modules in similar contexts.

It is important to note that most prehospital professionals in this region are women, as sex may impact learning styles, clinical confidence, and other relevant variables.^25^ ^,^ ^26^ Future studies are needed to assess the impact of gender on learning styles. It is also important to note that the data did not support any significant change in measured patient outcomes. This may be because measured outcomes were more impacted by issues in ED operations, such as staff boarding difficulties as well as limited capacity and resources. Also, there was a small decrease in ED and in-hospital mortality post-training, although the sample size was too small to detect any significant difference, and other interventions may have confounded the result.

Direct observation, prospective analysis, and improvement in methods are needed after such a training intervention to assess future patient outcomes. Overall, the promising association between a tailored training curriculum and improved post-test indicators in various trauma topics, as well as the acceptability of modules amongst participants, establishes the usefulness for such trainings amongst prehospital professionals. Further training should be considered for other groups including graduating medical students and general practitioners practicing emergency medicine.

Most importantly, this study is setting the stage for a longer term, comprehensive, and locally run emergency care training to sustainably establish knowledge retention and improve clinical process indicators.^16^

## LIMITATIONS

CHUK is a tertiary-care hospital, with multiple subspecialty training programs that were in their infancy at the time of this study. Other training programs could have introduced bias into the knowledge retention assessment and patient outcome analysis. Thus, these results may not be generalizable to other contexts. Training-specific limitations include interruptions in post-test administration due to leaves of absence of training staff, as well as loss of participants due to the need for staffing ambulances in critical need. As a result, their patient outcome data were not included in the study. The 11-month post-intervention analysis results did not have a control group, as equitable training was a necessary need at the time. More longitudinal studies evaluating the impact of tailored trainings on clinician retention and clinical effectiveness are needed.

Finally, process indicators to assess prehospital responsiveness did not encompass all skills from the training. Namely, the training improved prehospital professionals’ patient assessment skills, but clinical case scenarios encompassed much more than what could be measured from the predefined prehospital process indicator run sheet. Quantity of fluid administration could not be analysed as this was not documented, and the impact of the training on prehospital responsiveness to patients with burn injury, as well as hypoglycaemia, could not be accurately measured due to limited data available for this patient population.

## CONCLUSION

The study demonstrated that a basic course tailored to a group of prehospital EMS professionals in Rwanda could lead to improvement in emergency care knowledge, confidence, and an appropriate response to hypoxia. It is important to note that most prehospital professionals in this region are women. Context-driven education on prehospital emergency care is needed to appropriately address specific needs in LMICs and offers EMS professionals greater confidence in their knowledge and skills. The research contributes to the generalizable knowledge on prehospital care in resource-limited settings and serves as a foundation for the development of protocols that will improve patient care and strengthen the Rwandan healthcare system. Further studies are needed to evaluate such trainings in similar contexts, to investigate the impact on clinical outcomes.

### Dissemination of Results

The results from this study were disseminated to stakeholders including prehospital EMS professionals, the Ministry of Health, and hospital staff.
